# Uncertainty and Denial: A Resource-Rational Model of the Value of Information

**DOI:** 10.1371/journal.pone.0113342

**Published:** 2014-11-26

**Authors:** Emma Pierson, Noah Goodman

**Affiliations:** 1 Department of Computer Science, Stanford University, Stanford, California, United States of America; 2 Department of Psychology, Stanford University, Stanford, California, United States of America; Southwest University, China

## Abstract

Classical decision theory predicts that people should be indifferent to information that is not useful for making decisions, but this model often fails to describe human behavior. Here we investigate one such scenario, where people desire information about whether an event (the gain/loss of money) will occur even though there is no obvious decision to be made on the basis of this information. We find a curious dual trend: if information is costless, as the probability of the event increases people want the information more; if information is not costless, people's desire for the information peaks at an intermediate probability. People also want information more as the importance of the event increases, and less as the cost of the information increases. We propose a model that explains these results, based on the assumption that people have limited cognitive resources and obtain information about which events will occur so they can determine whether to expend effort planning for them.

## Introduction

When do we want to know? The question rises ubiquitous in an uncertain world, in manifestations both trivial–should you check the calorie count on that decadent croissant?–and momentous–should you learn whether you carry the Huntington's gene? [1]–[3]. Computing the value of information, 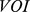, is thus a cognitive task of great importance. Classical decision theory offers one definition of 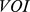: take the difference in expected utility of the optimal decision made with the information (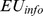) and the optimal decision made without the information (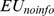) [4]. More formally, if the information is the value of a random variable 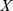 that takes values 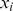 with probability 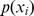, and our decision is which action 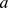 to take to maximize a utility 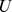 that is a function of 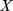 and 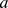, then 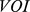 is the difference between the expected utilities with and without information:
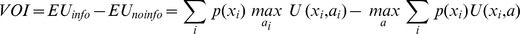




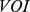
 is non-negative, which reflects the intuition that more information never makes your decision worse. Learning the croissant's calorie count might guide you to a salad instead [5]; learning you will not get Huntington's might make you delay a hasty marriage. The non-negativity of 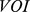 implies an important corollary: we should never 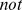 want information, and should be indifferent to it if and only if it cannot affect our decisions. As a description of human behavior, however, this proves false in two ways. On the one hand, people often desire information even when it will not affect their decisions: for example, women at risk for a mutation that predisposed them to breast cancer remained equally likely to get tested even when it would not affect their medical decisions [6]. People will delay a decision until they obtain information even when they would make the same decision regardless of the information [7]. Even animals sometimes display a preference for information even when it will not affect their decisions, possibly because of the same biological circuitry that motivates reward-seeking [8]. On the other hand, people are often strongly averse to information: they often do not want to learn that someone else got a better deal on an recent expensive purchase [9]; or the gender of their child before the birth [10]. While these deviations from classical decision theory have been dismissed as “irrational”, they indicate the model's inadequacy as a descriptor of human behavior.

Here, we explore one region that the classical model does not explain: we investigate a situation where information has no obvious effect upon decisions but people nonetheless care deeply about it. We ask people to imagine the following scenario: They are locked in a room for an hour. At the end of the hour, there is some chance they will gain or lose a sum of money. We then ask them if they would prefer to be told at the beginning of the hour whether they will gain or lose the money. There is no obvious decision to be made based upon this information: either way, people are locked in the room for an hour. Even if there are decisions to be made based on the loss or gain of money, they cannot be made until the person leaves the room, when they will have the information anyway. And yet people are not indifferent to the information.

We present a series of mathematical models that explain this lack of indifference. We begin with the classical model of a perfect Bayesian decision theorist. We then successively refine the perfectly rational model by adding a series of “empirically validated irrationalities” to our Bayesian agent. The first and most important refinement flows from our central thesis that because people have limited cognitive resources, they obtain information about whether events will occur so they can determine how to allocate cognitive resources to planning for them. The “decision” to be made here, which gives information its value, is a meta-cognitive one: how much effort to devote to planning for an event. Importantly, we believe that this goal of devoting the optimal effort to planning is a computational goal that guided the evolution of our cognitive processes, not necessarily a motive that people are consciously considering when they decide whether to obtain information. People may say, for example, that they wish to obtain the information because they feel nervous, but this nervousness is merely the brain's way of motivating them to pursue information in situations where it tends to be valuable for planning. (The fact that the same circuits are involved in both information-seeking and reward-seeking [8] also indicates the deep level on which information seeking is rewarded.) Put another way, our explanation operates on Marr's computational level [11], describing a goal of cognition; it does not necessarily operate on his algorithmic level, explaining the proximate means by which this goal is carried out.

We further refine our model using results from prospect theory [12]: first, people are risk averse, with concave monetary utility functions; second, people are loss averse, finding losses of money more psychologically important than gains; third, people perceive probability non-linearly, overestimating low probabilities and underestimating high ones. We then conduct two experiments testing the empirical predictions of these models.

## Models

We present a series of models for 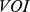 in the scenario described above: a person is locked in a room for an hour and is told there is a probability 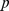 that they will lose or receive an amount of money 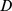 when they leave the room at the end of the hour (and a probability 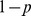 that nothing will happen). They can pay a cost 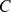 to learn at the beginning of the hour whether they will lose/receive the money. They indicate their desire for information on a 5-item Likert scale from “Strongly prefer not to know” to “Strongly prefer to know”. For each model, we link 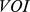 to the fraction of participants responding in each Likert category using an ordinal probit model [13]: the fraction of participants responding in Likert category 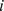 is 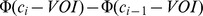, where 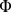 is the normal cumulative distribution function, 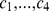 are parameters of the probit model, and 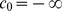, 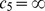 for convenience. We begin with the perfect Bayesian decision theorist model of 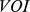 and successively extend it to produce four more realistic agents. After presenting the models, we explore how they fit the results of two experiments.

### Model 0: Perfect Bayesian agent

Classical decision theory predicts that
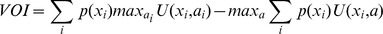
where 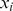 is an indicator variable that is one if the person will lose/receive the money and zero otherwise. Since there is no action that can be taken while in the room based upon this information that cannot be taken upon leaving it, the maximization over 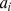 is trivial–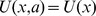
–and 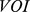 is zero.

### Model 1: Resource-rationality

Our most important refinement to the perfect Bayesian model flows from the basic observation that because people have limited cognitive resources, they obtain information about whether events will occur so they can determine whether to plan for them. If an event is not going to occur, there is no need to plan for it; if an event is going to occur, receiving this information at the beginning of the hour is advantageous because it gives you more time to plan.

We formalize this as follows. We assume that people can choose to devote computational resources 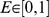 to planning for an event; if the event occurs, this planning pays off with utility gain 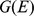, where 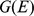 is monotonic increasing in 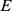 and 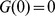. If the event has a probability 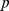 of occurring, the expected utility of planning for it is 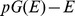: the possible gain of planning for it minus the certain cost of expending computational resources. Thus, a resource-rational agent who believes an event has a probability 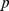 of occurring will devote 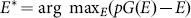 to planning for the event, with expected utility 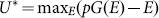.

We assume the agent has a choice between three options:

1. *Obtain information*: If the agent obtains the information, with probability 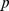 they find out the event will occur, plan for it, and gain utility 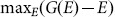; with probability 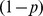 they find out the event will not occur, devote no effort to planning for it, and gain utility 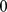. Thus, the expected utility of information is 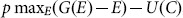, where 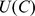 is the utility lost by paying 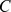 to receive the information and 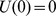.
2. *Live in uncertainty*: If the agent does not obtain the information and plans based on their belief that the event has probability 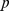 of occurring, they devote effort 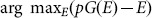 and gain utility 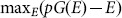. Note that if 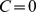, this option will be inferior to option 1 for 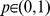.
3. *Live in denial*: If the agent does not obtain the information and does not plan, they do not have to devote resources to computing the maximization or to keeping the event in the back of their mind; because this devotion of resources has an opportunity cost, we assume the freeing up of computational resources has a utility 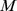.

**Figure 1:**
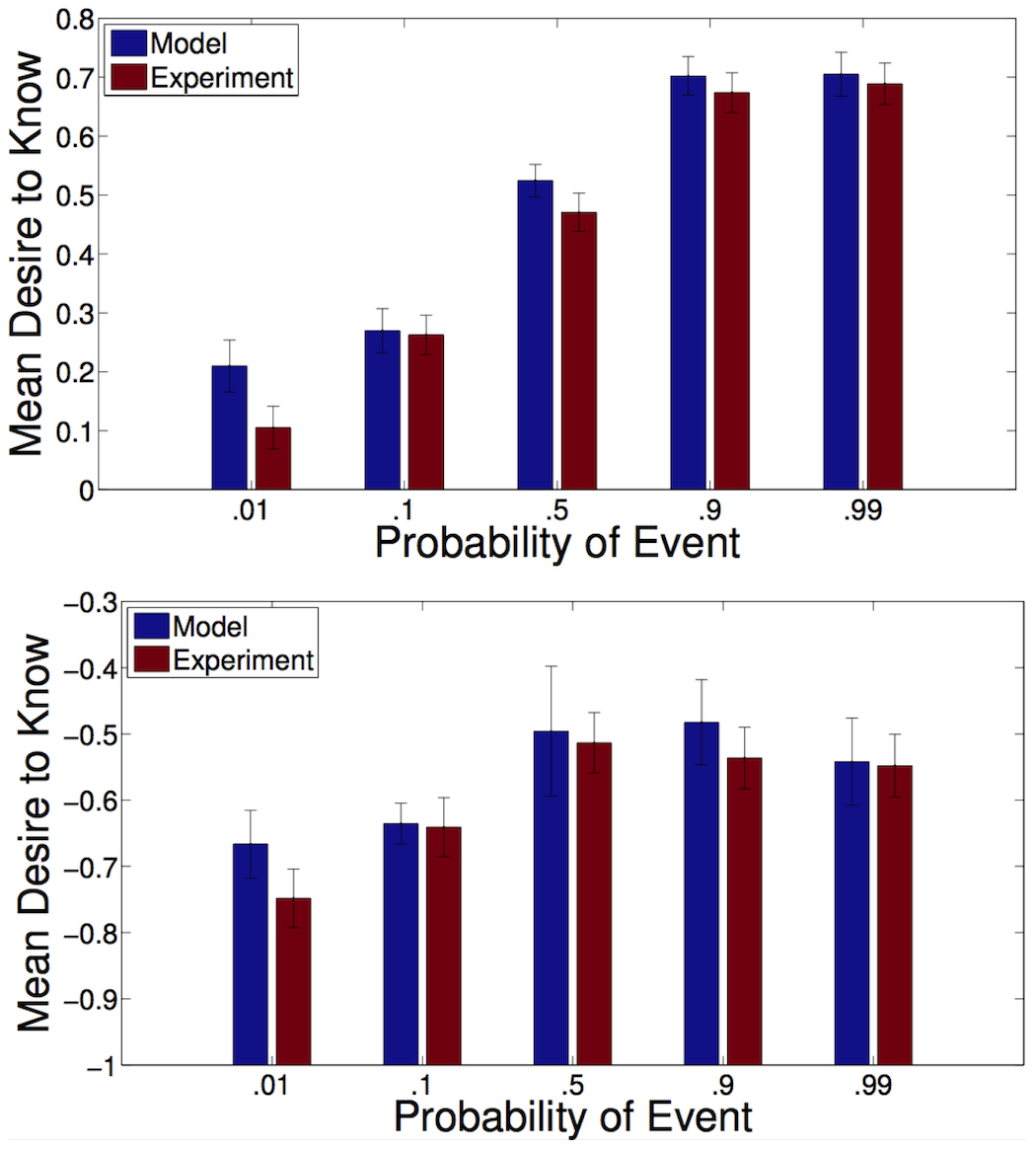
Desire for information increases monotonically in probability if information is costless (top), but exhibits a dropoff if information is not costless (bottom). The difference between the two trends is significant (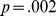, ANOVA). Top: Results of Experiment 1, costless information. Bottom: Results of Experiment 2, non-costless information. Vertical axis is mean response on a Likert scale that ranges from 2 (“Strongly prefer to know”) to −2 (“Strongly prefer not to know”). Model error bars are produced by bootstrapping samples and refitting each model; experiment error bars are standard error of the mean.

The value of information is the difference between the utility of (1) and the max of the utilities of (2) and (3):
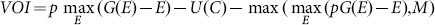



The intuition behind this model is simple. If information is free (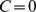), the only reason not to get it is because one does not want to plan for the event at all (“live in denial”). Since it makes the most sense to plan for events that are most probable, information grows more desirable, and denial less attractive, as the event grows more probable. On the other hand, if information is not free, there is another potential reason for not getting it: one still intends to plan, but the confirmation is too expensive (“live in uncertainty”). Thus, our model predicts that desire for information should increase essentially monotonically in probability if information is free but should exhibit a more pronounced dropoff if information is not free. This is in fact what we observe (Fig. 1).

We explore two methods of modeling the effect of varying the amount of money at stake, 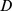. First, we assume that the utility of planning for an event scales with the importance of the event, since more important events are more important to plan for; thus, the utility of option (1) becomes 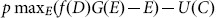, where 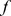 increases in the amount of money to be gained or lost, and the utility of option (2) becomes 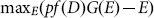. Second, we assume that the effort of planning for the event also increases with the importance of the event being planned for: it not only pays off more, but also requires more effort, to plan for one's approaching wedding than to plan for an approaching bus. Thus, the utility of option (1) becomes 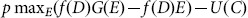, and the utility of option (2) becomes 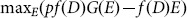. We refer to the former choice as “unscaled efforts” and the latter choice as “scaled efforts”; the latter fit our experimental data slightly better.

### Model 2: Risk aversion

While a risk-neutral agent would have a linear cost function [14], 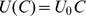, numerous authors have found that people are risk averse [15], so we refine our model by making 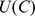 concave. Similarly, while a non-risk adverse agent would set 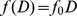, we use a concave 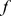. To restrict the space of models and prevent overfitting, we assume both 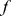 and 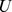 are logarithmic, a standard choice for monetary utility functions [16], setting 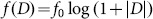 and 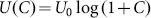.

### Model 3: Loss aversion

People are are loss-averse, finding losses of money more psychologically important than gains [17]–[19]. Therefore, we use 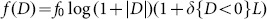 where 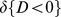 indicates whether the agent is losing or gaining money and 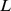 is a loss aversion parameter. (Note this adds an additional parameter to the model.)

### Model 4: Non-linear probability weighting

People's perceived probability of an event, 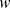, is non-linear in the actual probability of the event, 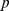: specifically, people sometimes overestimate the probability of unlikely events and underestimate the probability of very likely events. [20]–[22] use parameterizations of the form 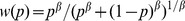, estimating similar values of 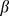 from experiments; we use the estimate in [17], 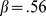.

## Experiments

### Experiment 1: Costless Information

For our first experiment, we set the information cost 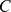 at zero (information was free) and varied the monetary amount to be gained or lost, 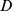, and the probability that the event would occur, 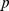.

#### Methods

Three hundred survey participants were recruited via Mechanical Turk and given the following prompt: “Imagine you will be locked in a room for an hour. At the end of the hour, you will be allowed to leave the room. There is a chance that something will happen to you when you leave. In each of the following situations, you will have to decide whether you want to be told at the beginning of the hour what will happen to you when you leave the room.” Participants were then given 30 situations of the form, “There is a [p] chance that [X]. Here is your choice: do you want to be told at the beginning of the hour whether [X], or do you want to wait until the end of the hour to find out?” The fourteen events X were “you will get D” or “you will get D taken away" where 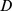 was $10, $50, $100, $500, $1,000, $5,000, and $10,000. The five probabilities 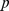 were 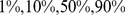, and 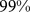. Participants answered using a 5-item Likert scale ranging from “I would strongly prefer 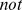 to be told” (scored as −2) to “I would strongly prefer to be told” (scored as 2). Each participant was given 6 randomly chosen X and for each X answered all five probabilities 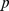 in random order. (Because subjects had no contact with experimenters, it is unlikely that they chose their answers merely to “appear rational” to experimenters.)

This research was reviewed for ethical treatment of human subjects by the Stanford University Administrative Panel on Human Subjects in Medical Research (IRB Number 349, Protocol ID 19974). It was determined to be in an exempt category, allowing the requirement for documented consent to be waived. Instead, participants provided consent by clicking “Start" after reading the following information: “By answering the following questions, you are participating in a study being performed by cognitive scientists in the Stanford Department of Psychology. If you have questions about this research, please contact Emma Pierson at emmap1@stanford.edu or Noah Goodman, at ngoodman@stanford.edu. You must be at least 18 years old to participate. Your participation in this research is voluntary. You may decline to answer any or all of the following questions. You may decline further participation, at any time, without adverse consequences. Your anonymity is assured; the researchers who have requested your participation will not receive any personal information about you." All participants provided consent by clicking through this page.

#### Results

**Figure 2:**
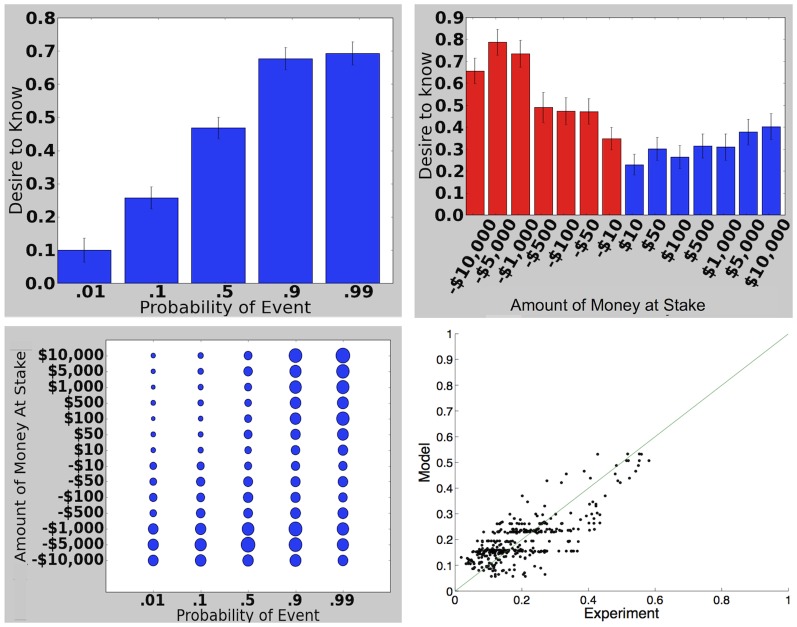
Costless Information, Experiment 1. Top left: Desire for information as a function of probability, averaged across all monetary amounts. Vertical axis is mean response on a Likert scale that ranges from 2 (“Strongly prefer to know”) to −2 (“Strongly prefer not to know”). Top right: Desire for information as a function of amount of money at stake, averaged across all probabilities. Bottom left: Desire for information for all pairs of probability and monetary amount. Larger dots indicate a greater desire for information. Bottom right: Modeled vs experimental values for all probabilities, monetary amounts, and Likert categories.

The first result of Experiment 1 was that people preferred to receive information even though there was no obvious decision to be made, a result not predicted by classical decision theory; the mean Likert scale response across all probabilities and monetary amounts was 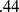, significantly different from 0 (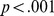, t-test). We observed three other significant trends (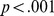, ordinal regression). People's desire for information increased (Fig. 2):

1. As the probability of the event increased
2. As the amount of money they might lose or gain increased
3. For losses as opposed to gains of money

These trends are consistent with, and extend, previous results. [23] found that people's desire to learn the outcomes of lotteries early was greater for losses as opposed to gains of money, consistent with trend (3). [24] found that if people were threatened with a shock and given the choice of listening to a informative tone that would warn them when the shock was about to occur or listening to music, they were more likely to choose the informative tone as the magnitude of the shock increased, consistent with trend (2).

Trend (1) is predicted by Models 1–4 though not by Model 0. If information is free, the only reason not to get it is to “live in denial”: if one intended to plan for the event, it would always make sense to find out for sure whether or not it would occur in order to devote the optimal resources to planning. For 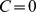 and 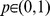, we have 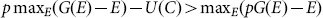, so “obtain information” is always preferable to “live in uncertainty”. Thus, the fraction of the population obtaining information is essentially the fraction for which 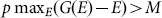, which increases monotonically in 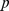. (As 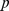 gets very close to 0 or 1, this no longer holds true, since the utility difference between “obtaining information” and “live in uncertainty” goes to zero, but this end behavior does not matter for most of the probability range, including that studied in our experiment.) Trend (2) is also predicted by Models 1–4 because more important events, involving larger sums of money, are more important to plan for. Trend (3) is predicted by Models 3–4, which incorporate loss aversion, making losses of money more important than gains.

We assessed the validity of our models by fitting them to our experimental results. Our parameters were 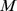, the utility of remaining in denial; 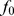, how quickly the utility of planning increased with the amount at stake; and 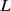, the loss aversion parameter, how much more important losses were than gains. (Note that Model 0 had no parameters besides the regression parameters of the probit model, Models 1–2 had 2 parameters, and Models 3–4 had 3). We chose a planning utility function 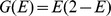, where 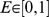, because it was concave (reflecting the diminishing marginal returns of additional planning); the results of the model were largely insensitive to choice of 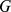.

The correlation 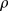 between fitted and modeled average responses on the Likert scale improved for each model, rising to.73 for Model 4. The most dramatic improvement, unsurprisingly, occurred between Models 0 and 1, reflecting the importance of the basic resource-rationality insight; the next largest gain was between Models 1 and 2, reflecting the importance of risk aversion.

**Figure 3:**
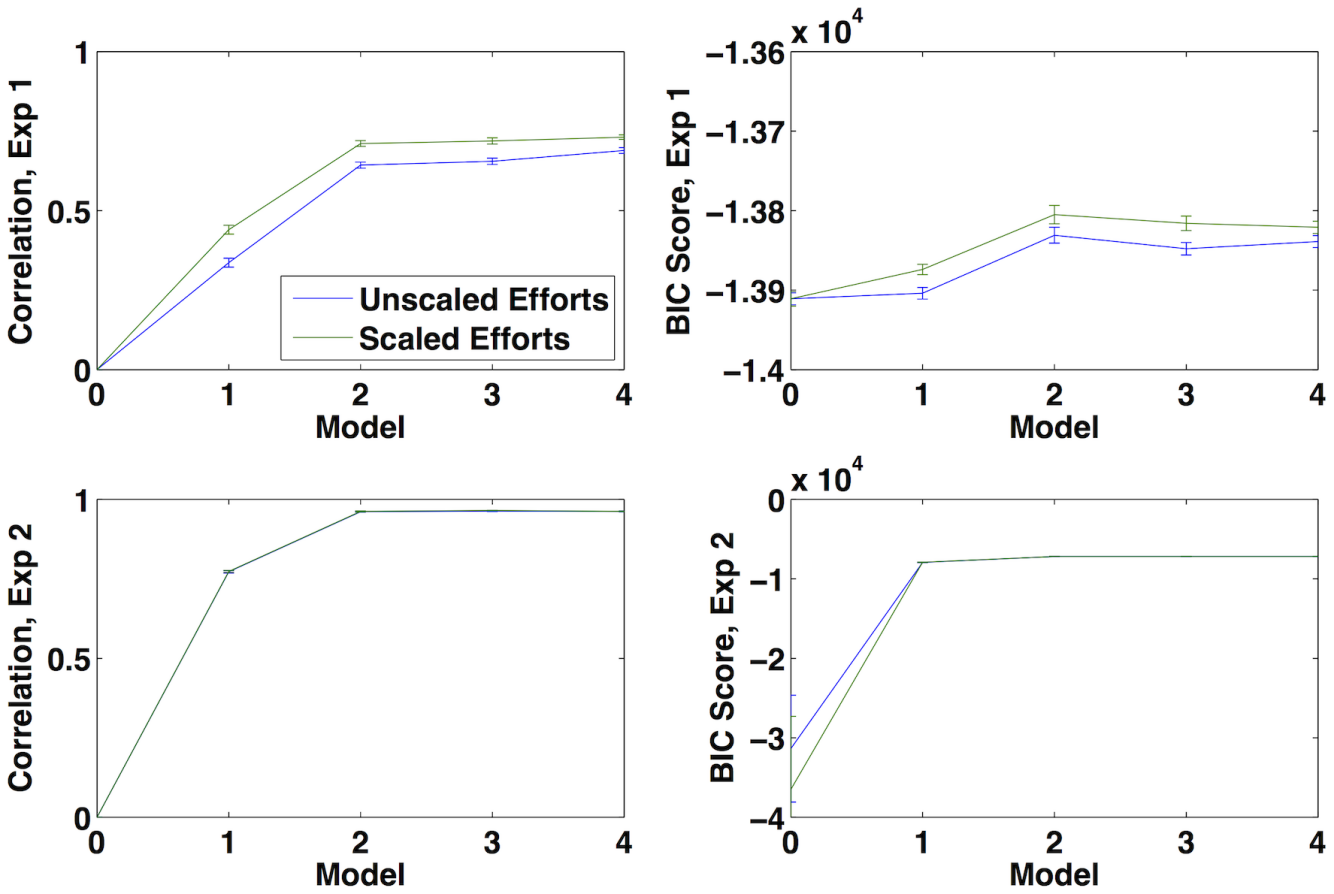
A comparison of all models by correlation with experimental values and BIC score. Model 2 has the highest BIC score in both experiments, with the most significant gains in both correlation and BIC score accruing between Models 0–2; the improvement is particularly dramatic in Experiment 2. Scaled efforts slightly outperforms unscaled efforts. Error bars are produced by non-parametric bootstrap.

**Figure 4:**
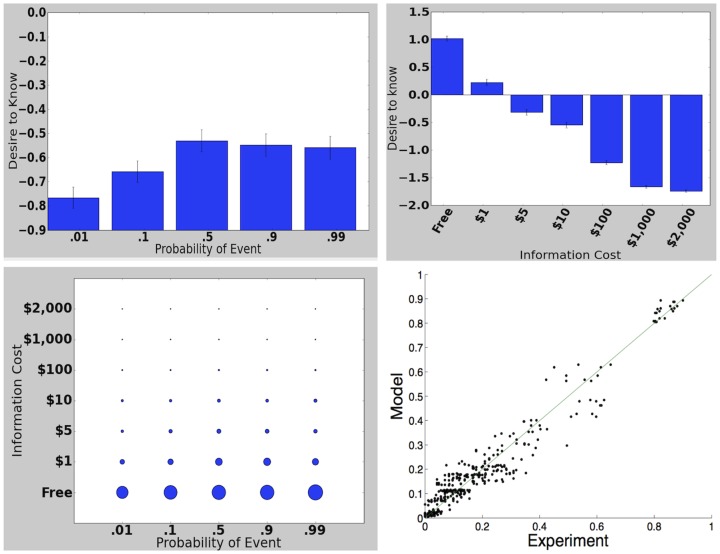
Non-costless Information, Experiment 2. Top left: Desire for information as a function of probability, averaged across all costs. Top right: Desire for information as a function of the cost of the information, averaged across all probabilities. Bottom left: Desire for information for all pairs of probability and cost. Bottom right: Modeled vs experimental values.

To select the best model (Fig. 3), accounting for the fact that later models used more parameters, we computed the likelihood of the data for each model and used the BIC score to penalize complexity: the score for each model was 
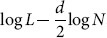
, where 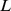 was the likelihood under the probit model, 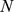 was the number of datapoints and 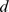 was the number of model parameters (including probit parameters). Model 2 had the highest BIC score (thus, all plots of model results in Figs. 1, 2, and 4 come from Model 2) substantiating the observation, discussed above, that the most important improvements to the perfect Bayesian model were resource-rationality and risk aversion, with loss aversion and non-linear probability weighting providing more marginal gains. The additional parameter added by Models 3–4 is penalized by the BIC score, explaining why these models were not favored; adding in the loss aversion parameter did improve the fit to the data, however, so our results are consistent with prospect theory.

### Experiment 2

For our second experiment, we varied the information cost 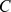 and the probability that the event would occur, 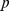.

#### Methods

The same prompt as in Experiment 1 was used, except participants were asked whether they would want the information if they had to pay $0, $1, $5, $10, $100, $1,000, or $2,000 at the beginning of the hour to learn whether they would gain or lose $1,000 at the end of the hour.

#### Results

We observed two trends (Fig. 4):

1. Desire for information decreased as the cost of information increased (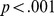, ordinal logistic regression).
2. Unlike in the first experiment, where desire for information increased monotonically in probability, if information was non-costless desire for information peaked at an intermediate probability. The discrepancy between this trend and the trend in Experiment 1 was significant (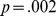, ANOVA). The decrease in desire to know between the 50% condition and the 90% and 99% conditions was also significant (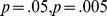, t-test). (Although the statistical significance is clear, the effect size was small, which should be kept in mind in interpreting the results.)

(1) is unsurprising; (2) is more interesting. Models 1–4 explain the discrepancy between probability trends for costless and non-costless information as follows: if information is free, people's only reason for rejecting it is if they choose to “live in denial”; if information is not free, people may prefer to “live in uncertainty” for high probability events and to “live in denial” for low probability events. Thus, the desire for information increases essentially monotonically for costless events (at least until 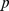 is very close to 1) but not for non-costless events (Fig. 1).

We fit our model using an ordinal probit model as described in Experiment 1. The fit for Models 2–4 was very good (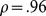). As in Experiment 1, Model 2 had the highest BIC score (Fig. 3), so all plots shown are for Model 2.

## Discussion

In this paper, we examine situations in which the classical decision theoretic model predicts that people ought to be indifferent to information because it cannot affect their decisions, but in which people nonetheless exhibit striking preferences. If information about whether an event will occur is costless, people prefer to receive it, with their desire increasing in the probability of the event and the amount of money at stake; if information is non-costless, people's desire for information peaks at an intermediate probability, showing a slight decrease thereafter, and decreases in the cost of the information.

We advance a series of models of how people value information that explain these trends as the classical decision theoretic model cannot. Like the classical model, we assume that people want to obtain information to make optimal decisions, but we extend the model in two ways. First, we broaden the sense of “decision” to include the meta-cognitive decision of how much effort to put into planning for an event. Second, we assume that people have limited cognitive resources, and that there is a cost to planning for or thinking about an event. Our results also provide another example of the utility of prospect theory, particularly the fact that people are risk-averse, with concave monetary utility functions; we also saw evidence of loss aversion and non-linear probability weighting, although these provided much more marginal gains.

This model provides a framework for understanding when and why people engage in the important cognitive task of obtaining information. It explains why they obtain information about whether an event will occur even when there is no obvious decision to be made–so they can determine how much effort to put into planning for the future in which the event happens–and also provides two reasons people have for rejecting information, which we refer to as the desires to “live in denial” or “live in uncertainty”. Living in denial is attractive when the probability of an event is low, and the most efficient allocation of cognitive resources is to not think about or plan for it at all; living in uncertainty is attractive when the probability of the event is near zero or one, and the residual uncertainty may more safely be ignored in determining how much to plan for an event. Living in denial may be preferable regardless of whether information is costless; living in uncertainty is only preferable if information is non-costless. Intuitively, if you are told that you have a 90% chance of having a deadly cancer, and there is a free test that will confirm whether you have it, your motives for rejecting the test would be very different than your motives for rejecting a test that costs $100,000. If you reject the free test, you are probably planning to live out your days in denial on the beach; if you reject the expensive test, you are probably planning to ignore the residual uncertainty and get chemotherapy.

One potential complication in interpreting our experimental results is that what participants 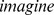 they will do may differ from what they will actually do. While it seems likely that the two are similar, what people imagine they will do is in itself important to observe, since many situations require us to imagine whether we would want information: for example, if you are entering into a non-exclusive romantic relationship, you may have to tell your partner ahead of time whether you would want to know if they are pursuing other people. Further, if what people imagine they will do deviates systematically from what they actually do, that in itself is a phenomenon worthy of more investigation.

In setting up our experiment, we chose a locked room because it provided a scenario in which the subject was clearly unable to act on the information, and we chose receipt or loss of money rather than some other event because it allowed us to manipulate the effects of increasing the amount of money. Future work might investigate related scenarios in which the reward or loss was altered or subjects were allowed to do something other than wait in a locked room. The latter question is particularly interesting because being locked in a room with nothing to do for an hour is a potentially aversive scenario, and subjects' reactions might change if they were allowed to distract or otherwise engage themselves.

We note that while both our experimental observations and our model are novel, they do represent an intriguing unification of previous work on why people choose to obtain information: the two motives our model predicts for information avoidance are both similar to previously postulated motives. [25] proposes a model that predicts that the value of information about whether an event will occur will increase as the event becomes more “salient”. All else being equal, higher probability events will be more salient (harder to ignore)–for example, people threatened with an electric shock have higher heart rates as the probability of the shock increases [24]–so this model may be thought of as predicting the monotonic increase in 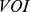 as a function of probability characteristic of the “live in denial” motive for information avoidance. Meanwhile, [26]–[28] have proposed analyses that measure 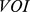 in terms of information theoretic measures like probability gain, KL distance, and Bayesian diagnosticity: these measures assign information the greatest value when it most reduces uncertainty, yielding the non-monotonic trend characteristic of the “live in uncertainty” motive. Both of these approaches are plausible, but neither can explain the whole of our results: our model thus provides a unification of previous work.

An alternate explanation for our experimental results comes from psychological expected utility theory [29], proposed by Caplin and Leahy, which extends Kreps and Porteus' dynamic choice theory [30]. Under Caplin and Leahy's model, people react to uncertainty with emotions like anticipation or anxiety and seek to maximize the utilities associated with these emotions. For example, a sports fan who is risk adverse might still place a bet on their favored team because doing so increases the pleasurable anxiety associated with watching the match. This model explains, for example, Lowenstein's finding [31] that people will pay more to delay (and thus anticipate) hypothetical kisses with movie stars, but do not want to delay events like electric shocks, which are unpleasant to anticipate. One might similarly try to explain our experimental results by speculating that people feel anxiety or anticipation associated with the potential gain or loss of money. Why then do we need our planning-motivated model? First, in order for Caplin and Leahy's model to make predictions, one must derive a form for the utilities associated with anticipation or anxiety; our model provides concrete predictions based on minimal assumptions. More importantly, saying that people feel anxiety or anticipation begs the question of 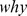 they feel these emotions. Our model provides the explanation: anxiety or anticipation are the proximate means by which our minds accomplish the ultimate goal of making us pursue information that helps us optimize planning. Put another way, our model provides an explanation on Marr's computational level [11], complementing Caplin and Leahy's explanation on the algorithmic level. We think it is entirely possible that some of our experimental subjects 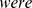 responding to anxiety or anticipation, not to a conscious desire to plan, but our model provides the ultimate explanation for why they felt these emotions and also makes concrete behavioral predictions.

More broadly, our model is another manifestation of the hypothesis that people are resource-rational: that is, they make decisions that are utility-maximizing given that cognitive resources are limited. This perspective has frequently proven useful in the past [32]: we are not perfect utility-maximizers, but agents struggling with limited resources to make the best sense we can of a complex and uncertain world. We constantly decide when to reduce that uncertainty by pursuing information, and those decisions will only become more important as information becomes more available. From our increasingly sophisticated ability to divine our pasts and futures from our genomes (do you want to know whether your father is really your father, or what you're likely to die of?) to the cornucopia of questionable internet services offering intimate information (do you want to know whose profiles your partner views, or how their past conquests rate them?) the value of information is as hard to determine as information is easy to obtain.
